# 

**Published:** 1852-07

**Authors:** 


### TO READERS AND CORRESPONDENTS.

Communications intended for publication, and all letters relating to the business of the Journal, or containing remittances in money, should be directed to Drs. Harris and Blandy, Baltimore, Md.

### *Medical and Dental Journals Received.*

1. *The Nashville Journal of Medicine and Surgery.* Edited by W. K. Bowling, M. D., assisted by Paul F. Eve, M. D., May, June, 1852. *In Exchange.*

2. *The American Journal of the Medical Sciences,* edited by Isaac Hays, M. D., Surgeon to Wills Hospital, &c., April, 1852. *In Exchange.*

3. *The New York Dental Recorder.* Edited by C. C. Allen, M. D., Dentist, April, May, June, 1852. *In Exchange.*

4. *The Dental Register of the West.* Edited by James Taylor, M. D., D. D. S. Published by order of the Mississippi Valley Association of Dental Surgeons. April, 1852. *In Exchange.*

5. *The Dental News Letter,* April, 1852. *In Exchange.*

6. *The London Medical Gazette.* March, April, 1852. *In Exchange.*

7. *Boston Medical and Surgical Journal.* Edited by J. V. C. Smith, M. D. April, May, June, 1852. *In Exchange.*

8. *The British and Foreign Medico-Chirurgical Review.* April, 1852. *In Exchange.*

9. *The Ohio Medical and Surgical Journal.* Edited by Richard L. Howard, M. D. April, 1852. *In Exchange.*

10. *The New York Journal of Medicine and the Collateral Sciences.* Edited by S. S. Purple, M. D. May and July, 1852. *In Exchange.*

11. *The Medical Examiner.* Edited by F. G. Smith, M. D., and John Biddle, M. D. April, May and June, 1852. *In Exchange.*

12. *The Southern Medical and Surgical Journal.* Edited by J. P. Garvin, M. D. April, May and June, 1852. *In Exchange.*

13. *The New York Medical Gazette, and Journal of Health.* Edited by D. M. Reese, M. D., L L. D. April, May and June 1852. *In Exchange.*

14. *The New Jersey Medical Reporter, and Transactions of the New Jersey Medical Society.* Edited by Joseph Parish, M. D. May and July, 1852. *In Exchange.*

15. *The Western Lancet and Hospital Reporter.* Edited by L. M. Lawson, M. D., and George Mendenhall, M. D. May and June, 1852. *In Exchange.*

16. *The North Western Medical and Surgical Journal.* Edited by J. Evans, M. D., and E. G. Meek, A. M., M. D. April, May and June, 1852. *In Exchange.*

17. *The Western Journal of Medicine and Surgery.* Edited by L. P. Yandell, M. D., and T. S. Bell, M. D. April, May and June, 1852. *In Exchange.*

18. *The New Hampshire Journal of Medicine.* Edited by Edward H. Parker, A. M., M. D. April, May and June, 1852. *In Exchange.*

19. *The Charleston Medical Journal and Review.* Edited by D. J. Cain, M. D. and F. Peyrel Porcher, M. D. May and July, 1852. *In Exchange.*

20. *The Buffalo Medical Journal.* Edited by Austin Flint, M. D. April, May and June, 1852. *In Exchange.*

21. *The Provincial Medical and Surgical Journal.* Edited by G. W. H. Rankin, M. D., and J. H. Walsh, Esq. April, May and June, 1852. *In Exchange.*

22. *The London Lancet*, Journal of British and Foreign, Surgical and Chemical Sciences, Chemistry, Literature and news. Editor Thomas Wakeley, M. D., for London. Sub-Editors, J. H. Bennet, M. D., and T. Wakeley, M. R. C. S. E. May, June and July, 1852. *In Exchange.*

23. *The Northern Lancet and Gazette of Legal Medicine.* A monthly Journal of Medical and General Science, Criticism, and Medical Jurisprudence. Edited by Horace Nelson, M. D., and Francis J. D'Avignon, M. D. May, June and July, 1852. *In Exchange.*

24. *The Dublin Medical Press.* April, May and June, 1852. *In Exchange.*

*Amalgam.*—We have been repeatedly charged with acting unjustly towards a few of our readers, who believe that amalgam may sometimes be used with advantage for filling teeth, in refusing to publish communications advocating its employment. We have done so, because we believed the subject had been fully discussed, and because we have never seen a tooth filled with this compound, that had not previously become a morbid irritant, that could not have been filled with gold. We are opposed to the use of it, under any and all circumstances, and yet, we admit two short articles in the present number from European correspondents, in which new methods of preparing it are described, but we cannot conceive that any particular advantage will be gained by these methods of preparation; at any rate, not sufficient to remove the objections that exist to the use of the compound. The unfavorable opinion which we expressed nearly twelve years ago, with regard to amalgam, has been fully confirmed by subsequent observation. Although we have not used it in our practice, scarcely a day passes in which we do not see teeth that have been filled with it, and found it necessary to replace it with gold.

*Bond's Dental Medicine.*—We are happy to learn that the first edition of this valuable work having been sold, a second is rapidly passing through the press, and will be published in a few weeks, by Messrs. Lindsay & Blakiston, of Philadelphia. We also learn that the work has been carefully revised and considerably enlarged.

### INDEX.

A. PAGE.

Arthur, Robert, treatment of Dental Caries, 3, 337, 505
Artistic Dentistry. By Dr. A. Hill, . . . 20
American Dental Literature. By Dr. R. Arthur, . 43
Artificial Teeth and dental operations in England, cheap, 101
Adjusting Forceps. By F. G. Colburn, . . . 113
Austrian remedy for cholera. 144
Am. Soc. of Dental Surgeons. 157
Artificial palate, velum, uvula, septum of the nose, alveolar process, and artificial tooth. By J. Linderer, . 258
Alveolar Abscess, and injury of the jaw. By C. Bew, 267
Allen, J., letter from, . 285
Anatomy of the Teeth, comparative, 286, 439, .600
Am. Med. Associations, transactions of, . . . 323
Address delivered before the Pennsylvania Society of Dental Surgeons. By Elisha Townsend, D. D. S., . 325
Attachment of single teeth to a plate by means of a silicious composition, . 328
Article for drying cavities in teeth praparatory to filling, 335
Address delivered before the graduating class of the Baltimore College of Dental Surgery, March 1st, 1852. By Eleazar Parmly, . 353
American Dental Patents, a historical sketch of. By Geo. W. Kendall, . 397
Antrum, spontaneous collapse of the walls of, . . 495
Allen's silicious cement, . 502
Artificial Teeth, a new base for, . . . 503
Address delivered before the Am. Soc. of Dental Surgeons at the annual meeting held in Philadelphia, August, 1851. By J. H. Foster, . . . 532
Amalgam Stoppings. By W. R. Bridgman, . . 561
Atmospheric Plates. By T. L. Buckingham, . . 651
Avery, John, illustrations of the successful treatment of cleft palate, . . 658

B.

Ballard, W. Charles, dental education, . . 59
Bew, C., alveolar abscess and injury of the jaw, . 267
Biddle, John, review of materia medica, . . 498
Beasley, Henry, pocket formulary, . . 499
Balt. Col. of Dental Surgery, annual commencement of, 500
Bridgman, W. R., on amalgam stoppings, . . 561
Bate, Spence, soldering apparatus, . . . 587
Buckingham, T. L., remarks on atmospheric plates, . 652
Balt. Col. of Dental Surgery, 672
Bond's Dental Medicine, . 672

C.

Caries of the Teeth, treatment of, . 3, 337, 505
Cancrum Oris. By J. L. Levison, . 107
Colburn, J. F. G., adjusting forceps, . . . 113
Canton, A, on teething, . 131
Craige, David, general and pathological anatomy, . 153
College of Dental Surgery contemplated, . . 332
Comparative Anatomy of the Teeth, . . . . 333
Cincinnati Med. Library Association, discourses delivered before the. By Daniel Drake, . . . 499
Cleft Palate, successful treatment of, By John Avery, 658

PAGE.

D.

Dental Education. By Chas. W. Ballard, . . . 59
Dental Surgery in the U. S., progress of, . . 92
Destruction of the dental pulp by the heat of electricity. By Thomas H. Harding, Esq., . . . 128
Dental Times, . . . 168
Drake, Daniel, a systematic treatise on diseases, &c., . 313
Dentistry, Philadelphia College of, . . . 330
Dental Surgery, New York College of, . . . 331
Davis, C. G., Dr. . . . 334
Dwinelle, W. H., on treatment of deep seated dental caries, . . . 442
Drake, Daniel, discourses delivered before the Cincinnati Med. Library Association, . . . 499
Dental Colleges and Dr. Trenor, . . . 501
Dental Operations, fees, reduced for, . . 504
Dumont, Dr., . . . 504
Downing, Toogood, relation of tic douloureux to disorders of the teeth, . 556
Dental inventions and pretensions, . . . 567
Dental Exhibition at the World's Fair, . . . 571
Dental Surgery, Ohio College of, . . . 671
Dwinelle, W. H., . . . 672

E.

Electro-galvanic process for depositing plates for artificial teeth. By A. Hill, D. D. S., . . . 38
Electric Heat, an instrument for applying in dental operations. By G. Waite, . 126
Exostosis, Orbital, . . 146
Epistaxis, instrument for arresting. By M. Gabriel, 147
Errata, . . . 335
Epulis, . . . 493

F.

Filling Teeth, over exposed nerve. By Chapin A. Harris, M. D., . . . 72
Foster, J. H. Annual Address before the Am. Soc. of Dental Surgeons. . 532

PAGE.

G.

Gold, new preparation for stopping teeth. By John Tomes, . . . . 115
Gold, impure, for dental purposes. By W. Imrie, . 140
Gabriel, M., instrument for arresting epistaxis, . . 147
General and Pathological anatomy. By David Craige, 155
Gilbert, Mr., and his perpendicular extraction, . 264
Gold, alloys of, for dental purposes. By James Robinson, . . . . 269
Gutta Percha, as a base for artificial teeth, . . . 332
George, late Mr., Durance, 335
Gratuitous services injurious. By Levison, . . . 590
Gardette, Dr., and his proposition again, . . . 669

H.

Hill, A., on artistic dentistry, 20
Hill, A., electro galvanic process for depositing plates, for artificial teeth, . . 34
Hawes, C. A., essay on professional empiricisms, . . 38
Harris, Chapin A., filling teeth over exposed nerve, . 72
Harding, Thomas H., destruction of the dental pulp. by the heat of electricity, . 128
Handy, W. R., mucous membrane of the mouth, . 169
Hamilton, Frank. H., destruction of the lower jaw, 306
Hemorrhage, from the extraction of teeth, excessive. By J. L. Levison, . . 367
Human Teeth, ten minutes advice on. By Horace Norton. . . . 500
Hunter's silicious cement, . 503
Handy, W. R., on patents in different branches of medicine, . . . 594

I.

Imrie, W., impure gold for dental purposes, use of, . 140
Intermarriage. By Alexander Walker, . . 150

PAGE.

Injury to the jaw following the removal of an incisor tooth, by the keyed instrument. By Thomas Underwood, Esq., . . 309

J.

Jordan, Henry, practical observations on the teeth, . 322

K.

Kendall, Geo. W., historical sketch of American dental patents, . . . 397

L.

Language for deaf mutes, . 156
Leslie, Allen, Mississippi Valley Association, report of, . 208
Linderer Joseph, artificial palate, velum, uvula, septum of the nose, alveolar process, and artificial tooth, description of, . . 258
Lower jaw, destruction of. By Frank. H. Hamilton, . 306
Levison, L. J., excessive hemorrhage from the extraction of teeth, . . 367
Levison on gratuitous services, . . . 590
Leslie, Dr. report of the proceedings of the Mississippi Valley Association of Dental Surgeons. By James Taylor, M. D., . . 381
Lower Jaw, anchylosis of. By Dr. Wernher, . . 496
Linn, John, importance of preserving the teeth, . 500

M.

Microscopist. By Joseph H. Wythers, M. D., . . 154
McGill College, annual announcement of, . . 154
Medical department, Washington University of Balto. 157
Medical appointment, . 168
Mucous membrane of the mouth. By Prof. W. R. Handy, . . . 169
Mississippi Valley Association of dental surgeons, report of. By Allen Leslie, 208
Moore, J. W., Queen's Hospital, Birmingham, . 489
Medical Sciences, Half Yearly Abstract of. By G. W. H. Rankin, M. D., . . 498
Meig's Velpeau's Midwifery. By W. Byrd Page, . 498
Mendenhall, Geo., Vade Mecum, . . . 499
Materia Medica, review of. By John Biddle, . . 499
Mechanical dentistry, . 504
Materials best adapted for filling teeth. By Wm. Robertson, Esq. . . 580

N.

Nitrate of silver, use of, in lacerated wound of the face, . . . 148
Norton, Horace, ten minutes advice respecting the human teeth, . . . 500

O.

Orbital exostosis, . . 146
Ohio College of Dental Surgery, seventh annual announcement . . 155
Osseous union of two temporary teeth, . . . 167
Ohio College of Dental Surgery, . . . . 168
Omission, . . . 168

P.

Professional empiricisms, by A. C. Hawes, . . 38
Professional and technical puzzle, . . . 149
Penn. Col., med. department, annual announcement of, 155
Perpendicular extraction. Mr. Gilbert and his Son . 264
Parmly, W. G., Dr. . . 333
Parmly, Eleazar. Address before the graduating class of the Baltimore College of Dental Surgery, March 1st, 1852, . . . 353
Page, W. Byrd, Meigs' Velpeau's Midwifery, . . 498
Pocket Formulary. By Henry Beasley . . . 499
Patents in different branches of medicine. By W. R. Handy, M. D., . . 594
Philadelphia College of Dental Surgery, . . 671

Q.

Quacks universally advertise, 166

PAGE.

Queen's Hospital, Birmingham. By W. J. Moore, Esq. . . . 489

R.

Remarks on gums of consumptive patients, peculiar appearance of. By T. Thompson, . . 138
Rush Med. Col., annual announcement of, . . 155
Resolution and argument. By E. Townsend, . . 183
Robinson James. Alloys of gold, . . . 269
Report to the Legislature of Mass. By A. Walker, . 324
Rankin, G. W. H., Half Yearly Abstract of the Med. Sciences, . . . 498
Robertson, Wm., on materials best adapted for filling teeth . . . 580

S.

Shepherd, S. M., Treatise on Dental Science and Practice, . . . 156
Snake, Joseph. physiology of the teeth, . . . 321
Sponge, gold, . . 334
Salivary fistula, . . 492
Silicious cement, Allen's, . 502
Silicious cement, Hunter's, 503
Soldering apparatus. By Spence Bate, . . 587

T.

Tomes, John, new preparation of gold for stopping teeth, . . . 115
Teething. By Mr. A. Canton, 131
Thompson, T., Remarks on gums of consumptive patients, peculiar appearance of, . . . . 138
Treatise on Dental Science and Practice. By S. M. Shepherd, . . . 156
Townsend, E., resolution and argument, . . 183
Teeth, comparative anatomy of . 286, 439, 600
Teeth, dissolving of, in solution of sugar, . . 311
Tumor, removal of, in the parotid gland, . . 312

PAGE.

Treatise, systematic. By Daniel Drake, . . 313
Teeth and their preservation. By Chas. Vasey, . . 321
Teeth, physiology of. By Joseph Snake, . . 321
Teeth, practical observations on. By Henry Jordan, . 322
Townsend, Elisha, Address delivered before Pennsylvania Society of Dental Surgeons, . . . 325
Taylor, James, on Dr. Lester's report of the proceedings of the Mississippi Valley Association of Dental Surgeons, . . . 381
Teeth, importance of. By John Linn, . . 500
Trenor, Dr., and Dental Colleges, . . . 501
Tic Douloureux, relation to disorders of the teeth. By Toogood Downing, . 556
Tartar on teeth, . . 577

U.

Underwood, Thomas, injury to the jaw, following the removal of an incisor tooth by the keyed instrument, 309
Urethra, stricture of. By Robert Wade, . 320

V.

Vasey, Charles, teeth and their preservation, . 321

W.

Waite, George. An instrument for applying electric heat in dental operations, . 126
Walker, Alexander. Intermarriage, . . . 150
Wythers, Joseph H. Microscopist, . . . 154
Wade, Robert. Stricture of the urethra, . . 320
Walker, A., report to the Legislature of Mass., . 324
Wernher, Dr., on anchylosis of the lower jaw, . . 496

